# Psychological Distress among Ebola Survivors Discharged from an Ebola Treatment Unit in Monrovia, Liberia – A Qualitative Study

**DOI:** 10.3389/fpubh.2016.00142

**Published:** 2016-07-04

**Authors:** Ionara Rabelo, Virginia Lee, Mosoka P. Fallah, Moses Massaquoi, Iro Evlampidou, Rosa Crestani, Tom Decroo, Rafael Van den Bergh, Nathalie Severy

**Affiliations:** ^1^Monrovia Project, Médecins Sans Frontières – Operational Centre Brussels, Monrovia, Liberia; ^2^Monrovia Project, Médecins Sans Frontières – Operational Centre Paris, Monrovia, Liberia; ^3^The Liberian-U.S. Joint Clinical Research Partnership/National Institute of Health Bethesda, Maryland, Liberia; ^4^Ministry of Health and Social Welfare, Monrovia, Liberia; ^5^Clinton Health Access Initiative, Monrovia, Liberia; ^6^Operational Department, Médecins Sans Frontières, Operational Centre Brussels, Brussels, Belgium; ^7^Medical Department, Médecins Sans Frontières, Operational Centre Brussels, Brussels, Belgium

**Keywords:** Ebola virus disease, Liberia, qualitative research, psychological distress, emergency response

## Abstract

**Introduction:**

A consequence of the West Africa Ebola outbreak 2014–2015 was the unprecedented number of Ebola survivors discharged from the Ebola Treatment Units (ETUs). Liberia alone counted over 5,000 survivors. We undertook a qualitative study in Monrovia to better understand the mental distress experienced by survivors during hospitalization and reintegration into their community.

**Methods:**

Purposively selected Ebola survivors from ELWA3, the largest ETU in Liberia, were invited to join focus group discussions. Verbal-informed consent was sought. Three focus groups with a total of 17 participants were conducted between February and April 2015. Thematic analysis approach was applied to analyze the data.

**Results:**

The main stressors inside the ETU were the daily exposure to corpses, which often remained several hours among the living; the patients’ isolation from their families and worries about their well-being; and sometimes, the perception of disrespect by ETU staff. However, most survivors reported how staff motivated patients to drink, eat, bathe, and walk. Additionally, employing survivors as staff fostered hope, calling patients by their name increased confidence and familiarity, and organizing prayer and singing activities brought comfort. When Ebola virus disease survivors returned home, the experience of being alive was both a gift and a burden. Flashbacks were common among survivors. Perceived as contagious, many were excluded from their family, professional, and social life. Some survivors faced divorce, were driven out of their houses, or lost their jobs. The subsequent isolation prevented survivors from picking up daily life, and the multiple losses affected their coping mechanisms. However, when available, the support of family, friends, and prayer enabled survivors to cope with their mental distress. For those excluded from society, psychosocial counseling and the survivor’s network were ways to give a meaning to life post-Ebola.

**Conclusion:**

Exposure to death in the ETU and stigma in the communities induced posttraumatic stress reactions and symptoms of depression among Ebola survivors. Distress in the ETU can be reduced through timely management of corpses. Coping mechanisms can be strengthened through trust relationships, religion, peer/community support, and community-based psychosocial care. Mental health disorders need to be addressed with appropriate specialized care and follow-up.

## Introduction

The West Africa Ebola outbreak was unprecedented. By 31st January, 2016, 28,646 individuals had been infected with the Ebola Virus, 17,323 of whom survived (1). While mortality rates were dramatic, communities and health systems were for the first time confronted with sizeable numbers of Ebola Virus Disease (EVD) survivors. In Liberia alone, more than 5,000 patients survived Ebola (1). Such survivors experienced a life-threatening event, and during their hospitalization, many were exposed to extreme suffering and were separated from their loved ones. Survivors from previous Ebola outbreaks have reported major barriers to resuming normal lives after release from treatment, such as emotional distress, health issues, loss of possessions, and difficulty regaining their livelihoods (2–4). In addition, Ebola survivors may face stigma or even violence (4–8). However, the challenges to reintegration of EVD survivors in society during the West Africa outbreak may have been very different to what was described previously, due to the sheer magnitude and the extensive media coverage of the outbreak.

In Monrovia, Liberia, Médecins Sans Frontières (MSF) managed an Ebola Treatment Unit (ETU), known as ELWA3. Survivors discharged from ELWA3 were provided psychosocial support. We conducted a qualitative study to improve the understanding of mental stress experienced by survivors during hospitalization and during subsequent reintegration in their communities. Furthermore, we explored the mechanisms that helped survivors cope with mental distress in order to better guide our supportive activities.

## Materials and Methods

### Design

Qualitative data were collected from EVD survivors: experiences of mental distress and coping mechanisms during hospitalization and reintegration in the community were explored. Data were collected through focus group discussions (FGD), allowing the researchers to explore multiple perspectives of reported experiences and views.

### Study Setting

Médecins Sans Frontières was one of the major actors to respond to the outbreak in Liberia, providing care for EVD inpatients in a number of ETUs in the country, including ELWA3 in Monrovia, with a capacity of 250 beds at the peak of the outbreak. MSF activities consisted of medical and psychosocial care for EVD inpatients. After discharge, medical and psychosocial follow-up for survivors was provided at home. Additionally, by the end of January 2015, an outpatient MSF survivor clinic was established to respond to the needs of ELWA3 survivors who reported physical and psychological complaints post-EVD. The survivor clinic provided comprehensive health care through medical consultations and individual and group counseling. When needed or requested, home visits were organized.

### Recruitment

Qualitative data were collected from EVD survivors, purposively selected from the survivors attending the survivor clinic. Survivors who felt comfortable to speak about their treatment and recovery were invited to join a FGD. Survivors who were receiving at least two counseling or medical consultations at the EVD survivor clinic were invited to join a FGD. Those interested were informed that participation was voluntary, and a transport allowance was provided. All the survivors received counseling.

### Data Collection

Focus groups were conducted between 6th February and 22nd April, 2015. Verbal-informed consent was obtained prior to the commencement of the FGDs. During verbal consent, participants were informed on the dynamic of the FGD’s criteria to be a participant, the concept of voluntary participation, confidentiality, the expected duration of the FGD (1 h), eventual risks, benefits (support better EVD patients and survivors), reimbursements (transport), dissemination of the results, and right to refuse or withdraw. Additionally, the research team informed that no patient names or identifying information will be used in any presentation or publication and that the notes, tapes, and transcripts will be kept locked in the MSF clinic and destroyed after 3 years.

To favor participation, groups were divided by gender. The first group included nine female survivors and was conducted in a tent used for psychosocial group counseling, outside the premises of the ETU. The second and third FGDs included male EVD survivors, and were conducted inside the survivor clinic. The second FGD included eight male survivors, of whom six were invited to a third FGD. An interview guide with open-ended questions was applied. There were four main themes: (1) mental health distress during treatment at ELWA3, (2) coping strategies to overcome mental health distress in ELWA3, (3) mental health distress after discharge from ELWA3, and (4) coping strategies after discharge from ELWA3. The three focus groups were conducted by one expatriate mental health adviser and three national counselors trained in qualitative research. In addition to hand-written notes, the FGDs were audio-recorded and analyzed.

### Data Analysis

An interim analysis was conducted showing that the experiences and views brought up were very similar across FGDs. Therefore, the researchers concluded that saturation was reached after three FGDs. From the data collected during the third FGD, no new codes and categories were generated. The FGDs were transcribed by the research coordinator. Any identifying information was removed to maintain anonymity. Two researchers employed a thematic analysis approach. Through inductive analysis, data were coded without applying a pre-existing theory or coding frame. A codebook was developed, and all data were coded. Clusters of linked codes were grouped into categories. The analyses from the different researchers were compared. In addition, generated categories were validated through triangulation with observations made during the regular work of both researchers, who provided psychological and mental health care to survivors in the clinic and the communities. When contradictions emerged, the researchers held discussions to develop a broader and deeper understanding of the data.

### Ethics

The study was approved by the University of Liberia-Pacific Institute for Research and Evaluation Institutional Review Board (Monrovia, Liberia).

## Results

### Mental Health Distress during the Treatment in Ebola Treatment Unit

#### Overwhelming Exposure to Death in the ETU

The daily exposure to the death of relatives, friends, and other patients was a very stressful experience and induced despair in all patients. To survive, patients had to eat, drink, and take medicines, while other patients died in front of them. Respondents commonly agreed this to be the worst experience inside the ETU.

I came in the ETU very sick and what struck me were the dead bodies. All people were dying in front of me, even my best friend. I was taking care of her when she died right in front of me while we were eating. While we were eating people passed by carrying dead bodies. (Female survivor, 28 years old)

Exposure to dead bodies was even more distressing as health-care workers could only periodically enter the ETU. Therefore, when patients died, their corpses remained often several hours among the living.

#### Concerns about Family Life outside the ETU

Ebola virus disease patients felt isolated from their relatives. Although patients could be visited in the family visit area, most patients reported that they did not receive visits. Multiple factors contributed to this. Family members did not know in which ETU their relative was admitted, or they had to take care of children or elderly in their homes, or they were too afraid to come to the ETU, or they did not have money for transportation.

Although feeling abandoned, patients could not forget the beloved ones they left behind in their homes, while they were struggling for their own lives. Therefore, the lack of contact with their family outside the ETU troubled hospitalized patients. Patients did not have news about the well-being of their family, while the ongoing deadly epidemic nurtured many rumors.

The governmental quarantine policy was another burden, as they knew that family of EVD patients were separated from the rest of the community and suffered stigmatization. Other uncertainties that distressed inpatients continuously included the economic situation of their families and the lack of information on who was replacing them as a caregiver for their children. In addition, patients knew that their family was not aware if they were alive or had died since they were admitted.

One thing that was killing us was flashbacks. Imagine taking treatment and thinking about what happened, behind you, the loss of your children, the quarantine of your family. (Male survivor, 44 years old)

#### Loss of Sense of Reality in the ETU

Some of the survivors described a sense of numbness and failure to connect with the reality inside the ETU, as if the physical and mental suffering within the ETU resulted in a reduced mental awareness. Somehow this state seemed to help some patients to reduce stress levels inside the ETU.

I had been working in a gold mine where people dressed with a mask before going into the water to dive, and when I see him coming with the mask on, it reminded me of the mine. The first 3 or 4 days I was there, to me it was like in the mine, or under the water, because when under the water, you have no feelings, you only feel warm, you urinate on yourself and only come out if you want to go to the toilet. (Male survivor, 55 years old)

Some survivors described how it took time after being discharged to recognize that their behavior inside the ETU was affected by all the death and suffering they had to face daily in the ETU. Only then they realized how their perception of reality and the conversations they had were not connected with reality.

(…) everybody died in the tent and now I think first I used to talk stupid things but now the people talk to me, so I can talk something with sense now. (Female survivor, 24 years old)

#### Ambivalent Views of ETU Staff and Care

The attitude from ETU staff was perceived ambivalently. Some survivors described the ETU staff as intrusive and not respectful. The patients described how staff threw things they had asked for, even talking harshly with them.

My stressful experience with staff was that whenever we requested for clothes, they threw it at us, whether we liked it or not. … Some workers were not treating us fair when giving us food. For example, they used to say: if you don’t eat your food and just sit there, your friends are dying, and you will soon die also. (Male survivor, 44 years old)

On the other hand, some survivors described the ETU staff as encouraging, as they motivated them to drink, eat, take a bath, and walk around. Patients also explained how being called by their name encouraged them, bringing a sense of identity, confidence, and familiarity.

In the ETU, a doctor called Dr XXX was with me almost every day. He gave me a lot of words of encouragement whenever he came in. He would call me by my name, stand close to me or sometimes sit with me on the bed and try to counsel me so I felt like home. From there I got very much motivated to take my pills and the Oral Rehydration Salts regularly, and I found myself recovering very fast thanks to his counselling. (Male survivor, 55 years old)

#### Coping Strategies in the ETU: Supportive Attitude from Staff, Peer Support, and Prayer

Three main coping strategies emerged from the FGD: supportive attitude from staff, peer support, and prayer. As highlighted above, the staff’s attitude was perceived differently by different survivors, though all agreed that a supportive attitude from the caregivers was encouraging when fighting EVD.

Furthermore, caregiver–survivors (peers) played a meaningful role. Wearing a lighter form of PPE (usually a blue suit), they could stay longer inside the ETU and, thus, develop a closer contact with the hospitalized patients. Having survived Ebola they brought hope to the minds of inpatients.

The nurses and counselors are very good, they really took care of me; …and the staff that used to be in blue suit used to help me [the caregivers in blue suits were EVD survivors working inside the ETU as peer counselors and auxiliary staff]. (Female survivor, 42 years old)

Finally, the presence of a religious leader, gathering patients to pray and sing together, was perceived by respondents as very supportive during the time they were admitted inside the ETU.

A community pastor came every morning and had devotion with us and prayed. That built up my strength. (Male survivor, 56 years old)

### Mental Health Distress after Discharge from the ETU

#### Loss of Beloved Ones

When EVD survivors returned home, the experience of being alive was both a gift and a burden. They came back from the dead but many had also lost their loved ones. Many survivors did not have a chance to mourn with their families or communities. When corpses were cremated, without funerals and formal burial, they did not even have a grave to visit.

We were nine in the house everybody died, I’m the only survivor, so I’m living but my spirit is not in my body, I can’t sleep. (Female 42 years old)

Additionally, some survivors lost their possessions and even their homes, when these were burned.

#### Stigma in the Community and Isolation Interfered with the Mourning Process

Upon their arrival back home, Ebola survivors reported they were still perceived as contagious Ebola cases and, thus, a threat for the community. Ongoing somatic complaints induced suspicion. Community members claimed that if they were still sick they could still infect the community. Messages from the government and organizations on Ebola transmission changed regularly, which nurtured confusion.

Some Ebola survivors were forced to divorce, were driven out of their houses, or lost their jobs as nobody wanted to buy their products at the market, or touch their money. Some survivors reported how their children refused to be fed by them and how relatives avoided them. They were prevented to go to religious meetings and public spaces, such as public restrooms and public water tabs.

When I was using the government toilet they were gossiping about me. Even when my children collect water, the people said “your mom got Ebola, your mom must not collect water here”. (Female survivor, 42 years old)

When rejected and ostracized from workplaces, community, church, and family, survivors felt isolated and humiliated.

“(…) I was feeling bad and up till now I’m still feeling bad when I left the ETU, because my man left me, because his other women said he must not come to me or else she will divorce him, up till now no understanding.” “(…) the place we were renting, they put us outside from the place, so we are living at my aunty place” (Female survivor, 24 years old)

My disappointment came from my workplace. I carry a written communication telling that I am back and free from Ebola and need a month to rest before starting my job. I was driven out by the chief of security … They called a crowd and said “Ebola survivor, go back home and don’t come here again”. I got very angry and left. (Male survivor, 39 years old)

Thus, survivors were often excluded from family and community life and could not return to their normal daily lives. Often “Ebola survivor” was used as a pejorative term. The subsequent isolation undermined their healing process and affected their ability to cope, as they received little support during their mourning and could not share their traumatic experiences.

#### Symptoms of Depression and Posttraumatic Stress Reactions

Suffering continued after discharge from the ETU. Not being able to pick up their routine again, their life was, thus, affected profoundly, and some started to question if there was a reason to stay alive. Besides depression, some survivors presented with posttraumatic stress reactions.

When I went home, I felt bad, because my two children died. I could not see them in the house again and the woman that carried me home was not able to talk to my family because I started crying. If I don’t take sleeping tablets I can’t sleep. (Female, 28 years old)

The very day, they brought me there, I was not myself, and dead people were all around me… when I’m sitting home my mind goes back to it … (Female survivor, 39 years old)

Flashbacks were very common among survivors, most frequently connected with the image of corpses inside the ETU. Triggers of flashbacks differed. For some it could be a person staring at them, for others flashbacks were triggered by seeing white boots (alike the boots worn by the ETU staff). Others had regular flashbacks appearing out of the blue.

For me, when I went home, the amount of people I saw dying used to come to my mind, it used to be playing on me. (Female survivor, 18 years old)

I am a different person in the group here (his own community). Sometimes people just stare at me and that gives me a flashback. What is it? Is it that I am going mad? (Male survivor, 55 years old)

#### Coping Strategies to Overcome Isolation and Stigma

To keep their minds busy after returning home, some survivors looked for work, or preferred to read novels or the Bible. Support of family, friends, faith, and participation in a survivor’s association were frequently mentioned coping strategies that supported Ebola survivors in dealing with mental distress. Some survivors wore their survivor status as a badge of honor, displayed resilience, and tried to educate family and community about Ebola. Especially male survivors were active in support groups.

When you have a problem, we sit down and see how best. Moving together with the survivors is where my happiness lies as survivor. (Male survivor, 33 years old)

The practice of health-care workers/health promoters accompanying survivors home after discharge was reported as supportive by some survivors. For survivors excluded from community life, psychosocial counseling and the survivor’s network were the only source of information and a helpful way to give meaning to life post-Ebola. Furthermore, these networks provided a space to process grief and mourn.

The help of the psycho-social group that kept visiting, giving me lecture, giving some words of consolation, helped to change my mood … Now I feel like any other person in the community. (Male survivor, 55 years old)

I love to go around with friends [other survivors] because I feel that if I associate with non-survivors I will be stigmatized. … the MSF psycho-social team … helped me a lot in uniting me with other family members [survivors], who are helping me today. (Male survivor, 39 years old)

## Discussion

One of the consequences of the largest-ever Ebola outbreak, in West Africa 2014–2015, was the unprecedented volume of Ebola survivors discharged from the ETU (1, 9). Being hospitalized in the ETU and facing stigma upon return to the home community were important sources of mental distress. Inside the ETU, the main difficulties were the daily exposure to corpses (frequency of entering the high risk zone was restricted for staff, thus corpses were removed only periodically) and the patients’ isolation from their families. Furthermore, the ETU staff’s attitude was sometimes perceived as rude and non-respectful, though the majority of survivors reported how the kind support from the MSF teams enabled them to cope with the daily stress inside the ETU. Distance between caregivers and patients may have been created by the strict infection control measures as well as the high workload for caregivers.

How to “humanize” care in the ETU? First, if feasible, inpatients should be exposed as little as possible to corpses. Witnessing the death of relatives, friends, and even health-care workers has been reported as a most harrowing experience (2). Therefore, deteriorating patients could be separated from other patients and receive intensified psychosocial care. Second, the practice of employing survivors as peer health-care workers should be emphasized: our results, supported by evidence from Sierra Leone (10) show how peer health-care workers can develop trusting and supportive relationships with inpatients. However, not all survivors may be mentally prepared to return to the ETU. Alternatively, interested survivors may play an important role in communicating outside the ETU to communities and family members of inpatients and the restoration of their own dignity by offering them the opportunity to contribute to the EVD control efforts. Third, to mitigate stress, continuous training and supervision of ETU staff (medical teams, hygienists, body management teams, survivors employed inside the ETU) on the psychosocial needs of inpatients may result in a more supportive environment. A recent study by Shultz (11) highlights the importance of psychological support for infected patients, their relatives, and their caregivers. Fourth, coping mechanisms of patients could be enhanced if inpatients could communicate regularly with their relatives outside the ETU and, thus, overcome isolation, an important stress factor (2). Designated meeting rooms with transparent dividers may allow visits of relatives inside the ETU (12).

For most survivors, the suffering did not end after discharge from the ETU. Many were excluded from social and professional life in the community. Already in 1986, Folkman et al identified communication, social support, positive thoughts and self-esteem, humor, meaning-focused coping (giving meaning to stressful experiences), and search for new activities as important coping mechanisms (13). However, these coping mechanisms were often denied by the survivor’s environment. Thus, mental distress was not relieved, and continuous mental distress may impede the waning of posttraumatic stress reactions.

In addition, months after discharge, many survivors may have presented a post-Ebola syndrome, which can involve joint pain, muscle pain, fatigue, eye problems, and hearing problems, and can necessitate specialized health care (14). Stigmatization and mental health conditions, such as depression and posttraumatic stress reactions, may amplify post-Ebola syndrome. Conversely, somatic complaints prevent survivors from resuming their daily routine and work, increasing their vulnerability, and foster community mistrust about their health status and infectiousness (15).

Survivors reported coping better with distress in their communities when they received support from their family, friends, the survivor network, and MSF psychologists, and counselors. Meetings with neighbors and community leaders may result in a tailor-made support plan for survivors (9). Health-care workers can play an important role and may be able to facilitate the reintegration. However, health promotion and psychosocial support teams often use a one-size-fits-all approach, providing general information about Ebola and fail to create a personal support network for each survivor. Survivors could inform future psychosocial programs. Survivor-led community-based approaches may foster social and peer support for survivors and their relatives (16). Furthermore, key community stakeholders may be capacitated to identify and refer survivors who present mental or physical health problems for medical follow-up.

This study had some limitations. First, due to the emergency context and multiple responsibilities/high workload for the researchers, it was not possible to collect data extensively. Although only three FGDs were conducted, the experiences and views brought up were very similar across FGDs, suggesting consistency across the data collection. In addition, the researchers were able to triangulate the generated theory with the observations they made when providing psychological care for survivors in the survivor clinic and communities. Second, the findings cannot be generalized: the coping mechanisms described here are specifically connected with the local culture, in which often a religious meaning is attributed to life events (17).

In conclusion, Ebola survivors from ELWA3 were exposed to death in the ETU and stigma in the communities. These traumatic events may induce posttraumatic stress reactions and depressive symptoms. To reduce distress among ETU inpatients, future Ebola control programs should consider a timelier and more appropriate management of corpses inside the ETU, private rooms for inpatients, utilize survivors as peer-support providers where possible, assure that all ETU staff are trained in psychosocial support, and facilitate better and more regular communication between inpatients and their relatives and family members. Coping mechanisms can be strengthened through trust relationships, religion, peer/community support, and community-based psychosocial care.

## Author Contributions

IR wrote the first draft of the paper. All coauthors contributed to the subsequent drafts. All authors have read and approved the final paper.

## Conflict of Interest Statement

The authors declare that the research was conducted in the absence of any commercial or financial relationships that could be construed as a potential conflict of interest.
